# Subcallosal area 25: Its responsivity to the stress hormone cortisol and its opposing effects on appetitive motivation in marmosets

**DOI:** 10.1016/j.ynstr.2024.100637

**Published:** 2024-05-01

**Authors:** Rana Banai Tizkar, Lauren McIver, Christian Michael Wood, Angela Charlotte Roberts

**Affiliations:** Department of Physiology, Development and Neuroscience, University of Cambridge, Downing Street, Cambridge, CB2 3DY, UK

**Keywords:** Anxiety, Depression, Anhedonia, Stress, Subcallosal anterior cingulate cortex, Area 25

## Abstract

Aberrant activity in caudal subcallosal anterior cingulate cortex (scACC) is implicated in depression and anxiety symptomatology, with its normalisation a putative biomarker of successful treatment response. The function of scACC in emotion processing and mental health is not fully understood despite its known influence on stress-mediated processes through its rich expression of mineralocorticoid and glucocorticoid receptors. Here we examine the causal interaction between area 25 within scACC (scACC-25) and the stress hormone, cortisol, in the context of anhedonia and anxiety-like behaviour. In addition, the overall role of scACC-25 in hedonic capacity and motivation is investigated under transient pharmacological inactivation and overactivation. The results suggest that a local increase of cortisol in scACC-25 shows a rapid induction of anticipatory anhedonia and increased responsiveness to uncertain threat. Separate inactivation and overactivation of scACC-25 increased and decreased motivation and hedonic capacity, respectively, likely through different underlying mechanisms. Together, these data show that area scACC-25 has a causal role in consummatory and motivational behaviour and produces rapid responses to the stress hormone cortisol, that mediates anhedonia and anxiety-like behaviour.

## 1 Introduction

One brain region repeatedly reported to be overactive in depression and anxiety is the caudal subcallosal cingulate cortex (scACC; Drevets et al., 2008; Hamani et al., 2011). Consistent with this is the finding that reduction of activity within this area, regardless of treatment modality (Fox et al., 2012; Mayberg et al., 2000; Siegle et al., 2012), coincides with successful outcomes. Moreover, intervention studies in marmoset monkeys provide evidence for the causal role of overactivation of area 25 within caudal scACC (scACC-25) in the symptoms of depression and anxiety. Specifically, scACC-25 overactivation induces anticipatory and motivational anhedonia (Alexander et al., 2018), a major symptom of depression and especially common in treatment resistant patients (Kelly et al., 2022; Słupski et al., 2020). It also heightens reactivity to uncertain threat, causes generalisation of conditioned threat responses and increases sympathetic cardiovascular activity alongside reduced heart rate variability (Alexander et al., 2020); symptoms commonly reported in generalised anxiety disorder (Hur et al., 2020; Pittig et al., 2013). This raises the question, what natural conditions or contexts is scACC-25 activated to heighten reactivity to threat and blunt anticipatory and motivational approach responses?

One likely candidate is stress. For example, area 25/14 scACC activity has been shown to correlate positively with plasma levels of the stress hormone, cortisol, regardless of behavioural context in macaque monkeys (Jahn et al., 2010). Moreover, scACC-25 expresses high levels of both mineralocorticoid (MR) and glucocorticoid (GR) receptors (Patel et al., 2000) making it sensitive to stress-mediated effects including circulating cortisol. However, the relationship of scACC-25 with stress is likely to be a complex one, since not only is scACC-25 responsive to cortisol but through its projections onto the hypothalamus (Joyce and Barbas, 2018) can regulate levels of cortisol release. To address this issue, the present study determined the effects of infusing cortisol directly into scACC-25. It was predicted that cortisol may induce a similar effect in scACC-25 as overactivation, consistent with the hypothesis that stress induces scACC-25 activation. Accordingly, we assessed its effects on both reactivity to uncertain threat, as measured with the human intruder paradigm and anticipatory appetitive arousal as measured by Pavlovian, discriminative conditioning (experiment 1).

The second issue this study was designed to address was the relative contributions of scACC-25 to the regulation of appetitive and threat-related behaviours. Previously, Wallis et al. (2017) showed that inactivation of scACC-25 in marmosets reduced conditioned threat responses, enhanced parasympathetic cardiovascular activity and increased heart rate variability, opposing effects to that seen following overactivation (Alexander et al., 2020). In contrast, inactivation appeared to have no impact on responsivity to rewarding stimuli as measured by Pavlovian conditioned appetitive responses (Alexander et al., 2018), suggesting limited basal activity during reward anticipation, despite the recent macaque data that indicates punctate scACC-25 firing during reward anticipation (Young et al., 2023). To determine whether scACC-25 is involved in the regulation of other aspects of appetitive responding beyond anticipatory arousal, experiment 2 of the present study determined the effects of inactivation on appetitive motivation, as measured by a marmoset's willingness to make progressively more responses for reward, and preference and appetitive consumption, as measured by the sucrose preference test (experiment 2). For both experiments marmosets received surgery to implant intracerebral cannulae targeting scACC-25, and following suitable recovery received localised scACC-25 microinfusions of varying concentrations of cortisol (experiment 1) and a cocktail of GABA_A_ and GABA_B_ receptor agonists for inactivation purposes and dihydrokainic acid (DHK, glutamate reuptake inhibitor) for overactivation purposes (experiment 2).

## 2 Material and methods

### 2.1 Subjects

Twelve marmosets (*Callithrix jacchus*, 6 females), bred on-site at the University of Cambridge Marmoset Breeding Colony, were housed in male/female pairs (males were vasectomized). Four subjects (2 females) were in Experiment 1 while eight (4 females) were in Experiment 2. All marmosets were naïve at the start of Experiment 1. Four marmosets were naïve (s1-s4) in Experiment 2. The remaining four had all previously received infusions to either activate or inactivate scACC-25 (s5 (Alexander et al., 2020); s6-8 (Alexander et al., 2018)). Data on scACC-25 overactivation on appetitive motivation has been reported previously (Alexander et al., 2018); although only total responses had been presented. For full details refer to Table 1.

**Table 1:**
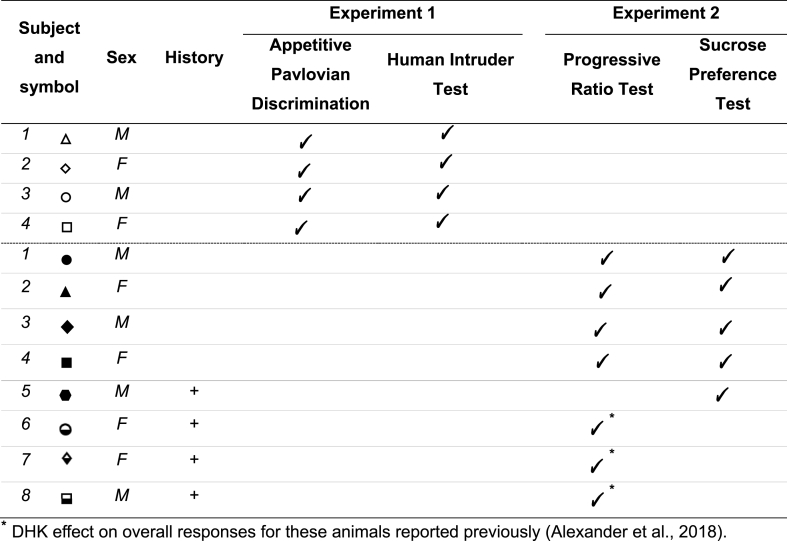
Summary of subjects, their symbol, prior experimental history, and experiments in which they participated.

* DHK effect on overall responses for these animals reported previously (Alexander et al., 2018).

Marmosets were housed in cages (280 × 120 × 98 cm) containing a nest box and a variety of environmental enrichments such as suspended ladders, wooden branches and ropes (described extensively in Alexander et al. (2018)). Marmosets were provided water ad libitum except for s1-4 during the progressive ratio testing phase where water bottles were removed at 17:30 Sunday-Thursday, and returned at 12:00 daily. Marmosets were provided with a modified diet on Sunday-Thursday consisting of MP.E1 primate diet (Special Diet Services, UK) and two carrot slices, otherwise, their diet was supplemented with fruit, rusk, malt loaf, and eggs. The room temperature and humidity were tightly controlled, with a 12-h light-dark schedule (7am lights on).

All procedures were carried out in accordance with the UK Animals (Scientific Procedures) Act 1986 and the University of Cambridge Animal Welfare and Ethical Review Body. All efforts were made to minimise animal suffering, to reduce the number of animals used, and to utilise alternatives to in vivo techniques, if available.

### 2.2 Surgical procedures

All subjects received chronic cannulae implanted into scACC-25 at coordinates +14.0 A P; ±0.7 L M with in-situ adjustments if required (Roberts et al., 2007; Stawicka et al., 2020). Four subjects (experiment 1) received telemetry probe implants for the transmission and recording of real-time cardiovascular activity. Marmosets were premedicated with ketamine hydrochloride (0.1 mL of 100 mg/mL solution, IM, Vetalar) and the nonsteroidal anti-inflammatory analgesic meloxicam (0.075 mL of 20 mg/mL solution s.c., Metacam, Boehringer Ingelheim). Details of surgical procedures, including the induction of anaesthesia, implantation of cannulae and telemetry probe, as well as postoperative care and weekly cleaning of cannulae are similar to previous publication as described by Alexander et al. (2018).

### 2.3 Drug treatments

Sterile drug infusions into scACC-25 were carried out while an experienced helper held the marmosets gently in their hands. The procedure was also performed under sterile conditions where all the surfaces including the guide and the cement mount around the guide were cleaned with 70% isopropyl alcohol (Alcotip, Universal). Drugs were delivered bilaterally via sterile injectors (Plastic One, C235I/SPC) connected to 10 μl Hamilton syringes using PTFE tubing. A pump produced a constant rate of 0.5 μl/min for DHK (6.25 nmol/μl; Tocris, UK – pretreatment time of 10min) and two doses of cortisol (1.5 and 5 ng/μl diluted in 0.9% saline; Hydrocortisone hemisuccinate, Sigma-Aldrich – pretreatment of 8min), and a rate of 0.25 μl/min for the muscimol/baclofen cocktail (0.1 mM muscimol/1.0 mM baclofen; Sigma-Aldrich – pretreatment of 25min) for a total duration of 2 min. Following infusion completion the injectors remained in place for an extra minute to allow for adequate diffusion. The dose of 5 ng/μl cortisol was chosen based on the study by Koot et al. (2014), and the dose of 1.5 ng/μl was derived by log-scale reduction to explore lower doses. Subcutaneous injection of 20 mg/kg cortisol (Ash et al., 2018) (or 0.9% saline as control) was administered with a pre-treatment time of 15 min (summarised in Table 2). All subjects were tested in the morning with no or minimal variation in the timing of the testing for each subject across the study. The rapid emergence (0–7min) and disappearance (20–27min) of the cortisol effect post i.m. Injection (Strelzyk et al., 2012), falls within the window of nongenomic effects (the 8 min of pre-treatment time plus 8 min of testing). To acclimatise marmosets to peripheral injections and central infusions they received mock injections/infusions where the entire procedure was carried out but with no actual infusion - first ‘offline’ at a separate time of day from testing in the behavioural testing apparatus.and then ‘on-line’ occurring 8–25 min prior to testing. When performance on either Pavlovian approach (experiment 1) or progressive ratio (experiment 2) was unaffected experimental infusions began.

**Table 2:** Details of drugs used in these experiments, their route of administration, dose and pre-treatment time, which is the time interval between completion of infusion and the time test commences.

Drug Vehicle	Mechanism	Route	Dose	Pre-treatment time
*Cortisol Hemisuccinate* *Vehicle: saline* *Experiment 1*	*GR agonist*	*Central infusion* *Area 25*	*1.5*, 5 ng/μl *Rate of 0.*5 μl/min *for 2 min*	*8 min*
*Cortisol Hemisuccinate* *Vehicle: saline* *Experiment 1*	*GR agonist*	*Systemic s.c. Injection*	20 mg/kg *0.*8 mL/kg	*15 min*
*Dihydrokainic acid (DHK)* *Vehicle: saline* *Experiment 2*	*EAAT*_*2*_*inhibitor*	*Central infusion* *Area 25*	*1.*35 μg/μl *Rate of 0.*5 μl/min *for 2 min*	*10 min*
*Muscimol/baclofen (MB)* *Vehicle: saline* *Experiment 2*	*GABA*_*A*_*/GABA*_*B*_*receptor agonist*	*Central infusion* *Area 25*	*11.*4 ng/μl *muscimol* *0.*214 μg/μl *baclofen* *Rate of 0.*25 μl/min *for 2 min*	*25 min*

An EAAT_2_ (Excitatory amino acid transporter-2) inhibitor increases overall levels of glutamate at the synapse by reducing the amount of glutamate taken up by its transporter.

### 2.4 Behavioural testing

Both the appetitive Pavlovian (experiment 1) and progressive ratio tests (experiment 2) were carried out in a sound-attenuated apparatus. The testing apparatus was fitted with a house light (3 W), speakers and three cameras connected to Power Director software (Cyberlink). A telemetry receiver (Physiotel, Data Sciences International) was located under the floor of the chamber, with data recorded by Spike 2 software (Cambridge Electronic Design). Marmosets were trained on these tests before surgery until they showed stable performance. The human intruder (experiment 1) and sucrose preference (experiment 2) tests were carried out in the top right quadrant of the home cage, where the subject was divided from the partner and were carried out post-surgery only. All marmosets received a minimum of two weeks post-surgery recovery, before any behavioural testing recommenced.

#### 2.4.1 Human intruder test

**Fig. 1:**
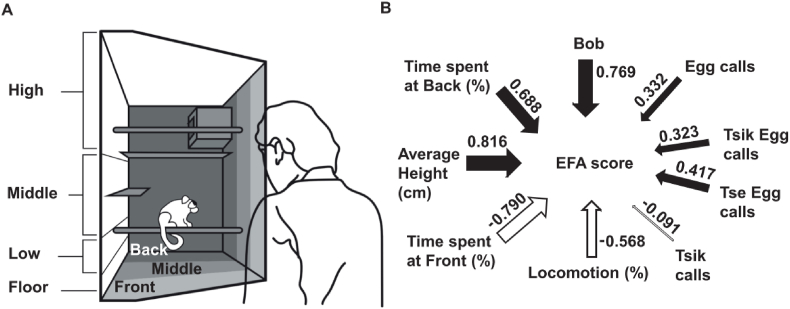
Human intruder behavioural paradigm. A. Human intruder test carried out in home cage illustrating zone categorisation. B. The factor loading of each measure used in the EFA score which is derived from an exploratory factor analysis from a cohort of 171 subjects (Quah et al., 2020).

The human intruder test was used to measure the intolerance of marmosets to uncertainty under experimental manipulations (described in detail in Quah et al., 2020). The test consists of three phases: separation, intruder, and recovery. Marmosets are initially restricted to the top right quadrant for 8 min (separation phase). An experimenter wearing a realistic latex human mask (Greyland Film, UK) and standard lab attire enters the room and stands in front of the home cage for 2 min and maintains constant eye contact with the subject (Intruder phase). The intruder then leaves and the marmosets behaviour is recorded for another 5 min (recovery phase). The subject's behaviour on the test, was recorded by a GoPro Hero 5 video camera on a tripod. Behaviour was scored offline (JWatcher software), measuring the time subjects spends in each zone (Fig. 1A). Their location, percentage time moving, head and body bobs and vocalisations (recorded by a shotgun microphone) were compiled into an exploratory factor analysis (EFA) score (Fig. 1B). Increase in this score indicates an increase in anxiety-like behaviour (Quah et al., 2020).

#### 2.4.2 Appetitive Pavlovian test

**Fig. 2:**
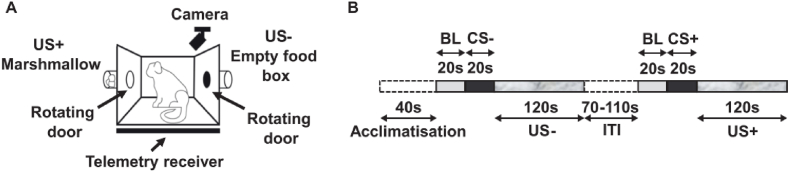
Appetitive Pavlovian paradigm. A. Schematic illustration of appetitive Pavlovian test apparatus with B. a timeline of a two-trial session used for all infusions.

For a full description of this test and the testing apparatus refer to Alexander et al., 2018, Alexander et al., 2021 (Fig. 2A). Briefly, prior to conditioning, all marmosets were habituated to the apparatus and reaching for freely available high incentive food (several pieces of marshmallow) in the food box. They were then habituated to the sight and sound of the food box door opening revealing reward. Once the subjects stopped showing a mild startle response (i.e., rearing and jumping) to the opening of the door, and started consuming marshmallow within 40s of its opening, they were advanced to conditioning sessions where two distinct tones (conditioned stimuli - CS) were played to cue reward (box full of marshmallow; CS+) and no reward, (empty food box, CS-) respectively. A biweekly testing schedule (10 sessions) was implemented Monday to Friday with animals receiving a CS + on only 5 of those sessions whilst at least one CS- (and a maximum of two) being presented on the majority of sessions. Minor variations of this schedule were used to prevent anticipation of rewarded trials. Each session started with a 40 s acclimatisation period and a variable inter-trial interval (ITI, 70–110s) in two trial sessions. In rewarded trials, access to a food box containing marshmallows (Unconditioned Stimulus, US+) was provided for 120s after a neutral sound cuing the reward (CS+) was played for 20s. In non-rewarded trials a neutral sound cued no reward (CS-) which was followed by display of an empty food box (US-) for 120s on the opposite side of the apparatus. Experimental manipulations occurred shortly prior to sessions containing a CS- followed by a CS+ (Fig. 2B). Anticipatory arousal for reward was measured by the number of rapid head rotations from side to side, called head jerks (Reekie et al., 2008), as well as raised mean arterial pressure (MAP) during the CS. For analyses, CS-directed data were calculated as the relative change in these measures compared to the 20-s baseline (BL) period prior to the CS period. The US-directed responses were analysed relative to the CS period (US minus CS).

#### 2.4.3 Progressive ratio test

Marmosets were first trained to touch the screen for reward, as described in full in Alexander et al. (2018). Briefly, subjects were first familiarised with the delivery of banana milkshake from a spout in the testing apparatus, and then trained to respond to a stimulus presented on a touchscreen for reward. The stimulus first was a green rectangle across the width of the screen and following successful touching to gain reward, it was changed to a square stimulus presented centrally and then to the left or right of the licker (Roberts et al., 1988). Once marmosets were reliably and accurately responding to this stimulus for a reward the stimulus was changed to a white circle presented at a fixed location (the animal's preferred side). Fixed ratio (FR) response schedules were then used to train the subjects to make repeated responses for reward with schedule progressing from FR1 to FR7 after which they were moved to a progressive ratio schedule (Pryce et al., 2004).

**Fig. 3:**
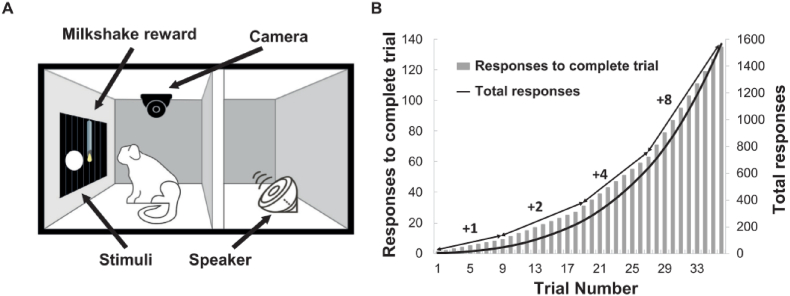
Progressive ratio behavioural paradigm. A. Schematic illustration of touchscreen-based test apparatus for progressive ratio test. B. Graph illustrating the progressive ratio schedule.

This test measured the motivation of marmosets to respond for reward (Fig. 3A). Marmosets pressed a circular stimulus on a touchscreen monitor to receive reward (5 s of milkshake (∼10% w/v banana Nesquik in whole milk) with a proceeding 0.5sec beep sound), with the number of required responses increasing across trials. The point at which marmosets stopped responding was termed the breakpoint. The required number of responses incremented by 1 for the first 9 trials, with this increment doubling every nine trials (Fig. 3B). Sessions terminated either after 30 min or after 2 min of inactivity.

Measures used in this test included.

• percent change in overall responses
• percent change in overall response rate

Manipulation data was always compared to the previous day's mock infusion data; thereby taking into account any variability of these measures between weeks.

• percentage of time spent licking the spout during non-rewarded periods
• post-reinforcement pause (PRP); the latency to respond to the stimulus immediately after a reward.
• average response rate per trial (RRPT); the number of responses made over the time between the first and last response.

To calculate the average PRP and RRPT for each subject the trial number of the shortest session of each subject across all manipulations was selected as cut-off point.

#### 2.4.4 Sucrose preference test

Sucrose preference test was used to measure the impact of scACC-25 manipulations on marmosets’ consummatory and preference behaviour, similar to Alexander et al. (2018), using 6% sucrose solution (w/v, Sigma-Aldrich). Marmosets were first habituated to the sucrose solution by introducing two identical 6% sucrose solution bottles in the upper right quadrant of the cage for 48 h. Subsequently, a marmoset was separated from their cage mate by dividing them off into the top right quadrant of the home cage with the nest box and usual water bottle removed. They were then presented with identical plastic bottles, one containing water and the other containing sucrose solution. Once marmosets achieved a stable sucrose preference of ∼80–90% over two sessions, the experimental manipulations took place.

Measurements included.

• total sucrose consumption (g),
• total water consumption (g),
• ratio of sucrose consumption over total consumption (sucrose preference).

For all behavioural tests, on the day of manipulation, the subject received the infusion/injection, then were put back in the home cage for the period of designated pre-treatment time depending on the type of drug. They were then taken to the testing apparatus or tested in the home cage. A minimum of one week was given between each manipulation, and the manipulations were counterbalanced among subjects when possible. No subject was omitted from any of these procedures.

**Fig. 4:**
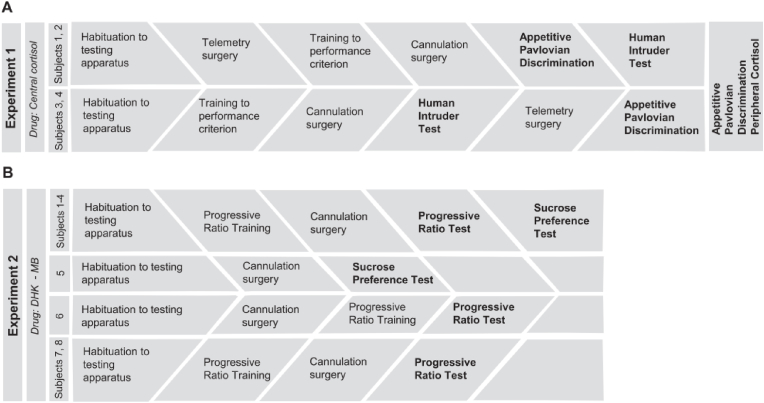
Flow chart demonstrating the order of experiments carried out in A. Experiment 1 and B. Experiment 2. In Experiment 1 (n = 4), half the marmosets (subjects 1 and 2) were tested on appetitive Pavlovian discrimination before the human intruder and vice versa for the other half (subjects 3 and 4). All subjects then proceeded to appetitive Pavlovian test and peripheral cortisol manipulation. In experiment 2, seven out of eight marmosets were tested on progressive ratio, four of which were also tested on sucrose preference. One animal was only tested on sucrose preference.

For a detailed description of tests and a summary of their order for all subjects refer to Fig. 4.

### 2.5 Statistical analyses

Statistical analyses were performed using IBM SPSS Statistics (version 28). In experiment 1, a one-way repeated ANOVA (3 levels: vehicle, cortisol 1.5 ng/μl, cortisol 5 ng/μl), and one-sample *t*-test (difference score between drug and vehicle) were used. For multiple comparisons Bonferroni corrections were applied and if no correction was used it was reported. In experiment 2, two-way mixed measures ANOVAs were used in progressive ratio test for three measures of total responses, response rate and licking outside reward delivery, using drug as a within subject factor (3 levels) and water restriction as a between subject factor (2 levels). A one-way repeated measures ANOVA (3 levels: vehicle, MB, DHK) was used in progressive ratio test in measures of average response rate per trial and post-reinforcement pause, as well as in sucrose preference test for measures of sucrose preference, sucrose, and water consumption. Wherever data transformation was necessary to meet the assumptions of ANOVA, the type of transformation is reported. For post hoc analyses Sidak correction was applied for pairwise comparisons. Whenever assumption of sphericity using Mauchly's test was not met Greenhouse-Geisser correction was applied.

### 2.6 Post-mortem assessment of cannulae placement

**Fig. 5:**
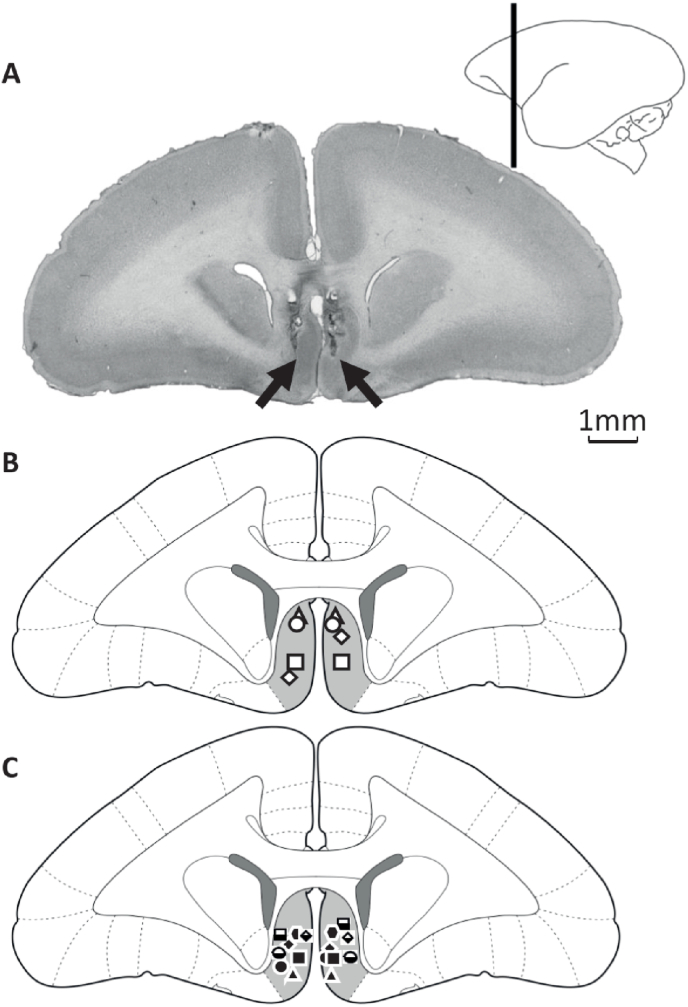
Histological verification of scACC-25 cannulation. A. Example of a cresyl stained coronal section with infusion site indicated by black arrows. B and C depict schematics indicating the confirmed infusion placements of subjects in experiments 1 and 2 respectively, within scACC-25 (light grey shading) at AP +13.80 (Paxinos et al., 2012). The range of infusion placements lay between AP 13.00–13.80.

Marmosets were premedicated with ketamine hydrochloride (0.1 mL of a 100 mg/mL solution, i.m.) and subsequently euthanised with sodium pentobarbital (Dolethal; 1 mL of a 200 mg/mL solution, i.v.). Following this, transcardial perfusion of 500 mL 0.1 M phosphate-buffered saline (PBS; Sigma-Aldrich) was conducted followed by 500 mL of 10% formalin solution. The brain was then extracted and placed in 10% formalin solution for 24 h, then 0.01 M PBS-azide solution for 48 h and finally 30% sucrose for 72 h. The brains were then cut into 40 μm coronal sections and mounted on gelatin-coated slides and stained with cresyl violet to confirm cannula placement. Sections were then visualised and photographed using a M205FA stereo microscope (Leica, UK). Histological assessment confirmed that subjects successfully had cannula placement into area 25 (Fig. 5).

## 3 Results

### 3.1 Experiment 1a: central infusion of cortisol increased anxiety-like behaviour in a human intruder test

**Fig. 6:**
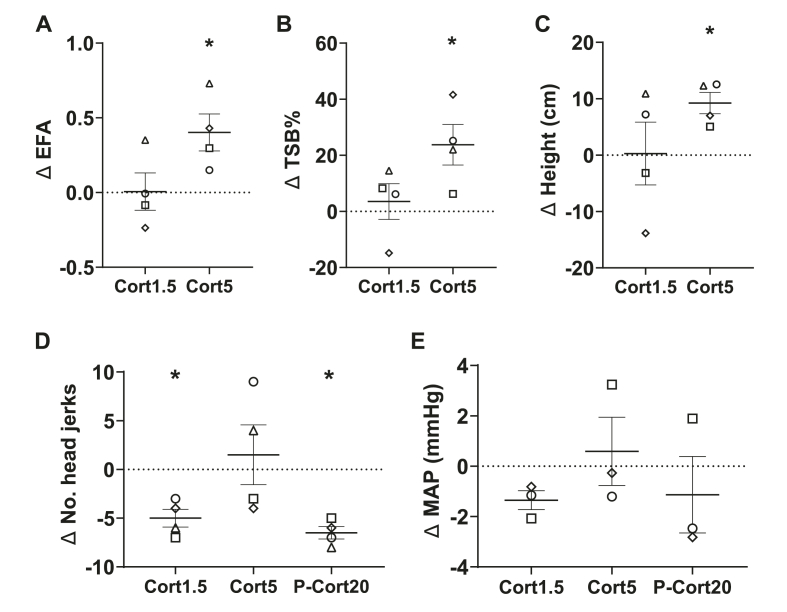
The effects of scACC-25 cortisol infusion on responsivity to uncertain threat and anticipatory arousal. A. Responsivity to an uncertain threat in the form of a human intruder was increased by scACC-25 infusion of Cort5 with an increase in the EFA score, whilst Cort1.5 had no effect. The factors contributing to this EFA increase were B. the time spent at the back of the cage (TSB%) and C. average height. Data are displayed as mean ± SEM error, *p < 0.05. D. Infusion of cortisol into scACC-25 decreased CS + directed head jerk behaviour at 1.5 ng/μl (Cort1.5) but not 5 ng/μl (Cort5) relative to saline. Peripheral injection of 20 mg/kg cortisol also decreased this behaviour (P-Cort20). E. Neither central or peripheral administration of cortisol impacted anticipatory cardiovascular arousal, with CS-directed MAP being unaffected. Female marmosets: diamond and square.

Infusion of cortisol into scACC-25 dose dependently heightened reactivity to a human intruder as revealed by an increase in the overall EFA-derived anxiety score (*F*_*(2,6)*_=*7.64, p*=*0.023, η*^*2*^=*0.717*). The increase in anxiety-like behaviour was only observed with cortisol 5 ng/μl (Fig. 6A; *t*_*(3)*_=*3.26, p*=*0.047 (not corrected), CI*=*[0.01, 0.79]*). This was a consequence primarily of an increase in avoidance of the intruder shown by their moving further back (Fig. 6B; *t*_*(3)*_=*3.28, p*=*0.046 (not corrected), CI*=*[0.71, 46.81]*) and higher (Fig. 6C; *t*_*(3)*_=*4.89, p*=*0.016, CI*=*[3.22, 15.23]*) in the cage. Cortisol 1.5 ng/μl did not impact any measure in this test (*EFA score p*=*0.963; TSB%, p*=*0.620; height, p*=*0.964*).

### 3.2 Experiment 1b: cortisol infusion into scACC-25 reduced anticipatory behavioural arousal in an appetitive pavlovian test

Infusion of 1.5 ng/μl cortisol (*t*_*(3)*_=*-5.48, p*=*0.024, CI*=*[−7.91, -2.09]*), but not 5 ng/μl (*t*_*(3)*_ = *0.49, p*=*0.658, CI = [−8.27, 11.27]*) into scACC-25 reduced anticipatory behavioural arousal to the CS+ (Fig. 6D and E), with neither dose impacting cardiovascular arousal (*1.*5 ng/μl*: p*=*0.069,* 5 ng/μl*: p*=*0.707*). Dampened behavioural arousal to the CS+ was also achieved by a peripheral injection of 20 mg/kg cortisol (*t*_*(3)*_=*-10.07, p*=*0.002, CI*=*[−8.55, -0.45]*) with cardiovascular arousal similarly unaffected (*p*=*0.532*). Importantly, both CS- and US + mediated responses were unaffected by either dose (*CS- 1.*5 ng/μl*: p*=*0.718,* 5 ng/μl*: p*=*0.718; US* + *1.*5 ng/μl*: p*=*0.467;* 5 ng/μl*: p*=*0.324*) consistent with previous findings with overactivation of scACC-25 (Alexander et al., 2018).

### 3.3 Experiment 2a: opposing effects of scACC-25 inactivation and overactivation on motivation

**Fig. 7:**
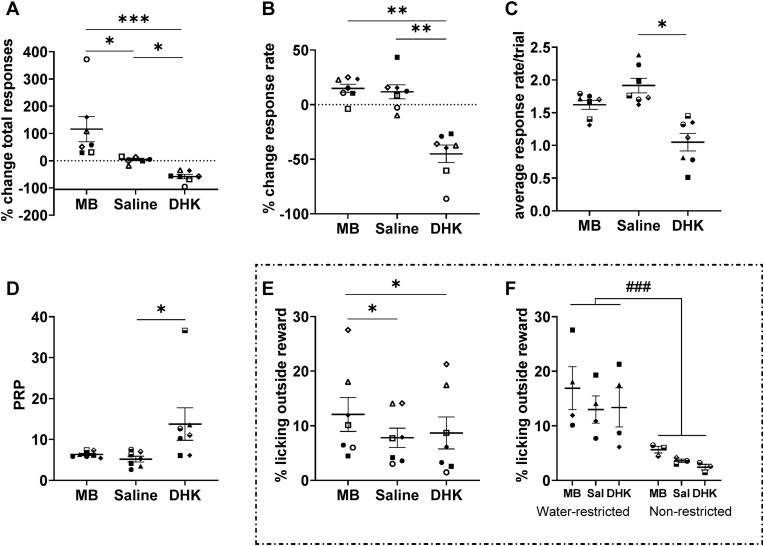
The effect of scACC-25 interventions on appetitive motivation in the progressive ratio test. A. Inactivation (MB) and overactivation (DHK) of scACC-25 led to increases and decreases in overall responses, respectively. B. DHK infusion into scACC-25 reduced overall response rate compared to saline and MB. C. Average response rate per trial was decreased by DHK compared to saline. D. DHK increased the post reinforcement pause (PRP) compared to saline. E. Inactivation of scACC-25 led to an increase in the amount of time spent licking the spout outside of reward delivery when compared to saline and DHK. F. Water restriction increased the time spent licking outside reward delivery regardless of the effect of drug manipulation. Data are displayed as mean ± SEM error, *p < 0.05, **p < 0.01 and ***p < 0.001. Female marmosets: filled triangle, filled square, open circle, open diamond.

Overall responses on the PR test were affected by drug manipulation (*F*_*(2,10)*_=*45.78, p*<*0.001, η*^*2*^=*0.88, cube-root transformed*), where MB-induced inactivation of scACC-25 increased (*p*=*0.048*) whilst DHK-induced overactivation of scACC-25 reduced (*p*=*0.011*) responding (Fig. 7A), with the between manipulation comparison also significant (*p*<*0.001*).

These effects were accompanied by changes in response rate (*F*_*(2,12)*_=*38.54, p*<*0.001, η*^*2*^=*0.92*) with a reduction following overactivation compared to saline (*p*=*0.002*; Fig. 7B), whilst inactivation had no effect compared to saline (*p*=*0.994*). Analysis of the two components that shape response rate, average response rate per trial (*RRPT; F*_*(2,12)*_=*12.36, p*=*0.001, η*^*2*^=*0.67*), and post-reinforcement pause (*PRP; F*_*(2,12)*_=*10.15, p*=*0.003, η*^*2*^=*0.63, log-transformed*) revealed that overactivation slowed both RRPT (*p*=*0.026*) and PRP (*p*=*0.032*), whilst MB-induced inactivation was without effect compared to saline (*RRPT, p*=*0.067; PRP, p*=*0.30,* Fig. 7C and D).

Inactivation saw an increase in licking outside of the reward delivery period (*F*_*(2,10)*_=*12.99, p*=*0.002, η*^*2*^=*0.72, log-transformed*; Fig. 7E and F). Inactivation increased ‘off target’ licking compared to both saline (*p*=*0.023*), and overactivation (*p*=*0.025*), whereas, overactivation itself had no significant effect (*p*=*0.540*). Water restriction in general led to increased licking outside reward delivery with a significant main effect of water schedule (*F*_*(1,5)*_=*20.51, p*=*0.006, η*^*2*^ = *0.80, log-transformed*) but it did not impact overall responses or response rate (*F*<*1* for all analyses).

### 3.4 Experiment 2b: inactivation of scACC-25 increased, whereas overactivation decreased, sucrose consumption

**Fig. 8:**
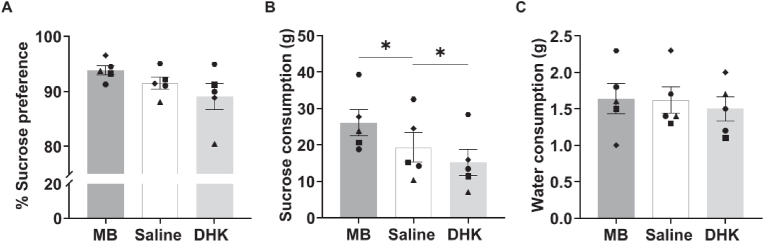
The effect of scACC-25 interventions on consummatory behaviour. A. Inactivation and overactivation of scACC-25 had no effect on sucrose preference. B. Inactivation of scACC-25 increased consumption of sucrose solution compared to saline whereas DHK decreased it. C. Neither drug manipulation had any effect on water consumption. Data are displayed as mean ± SEM error, *p < 0.05. Female marmosets: triangle and square.

There was no drug effect on sucrose preference (*F*_*(1.06, 4.23)*_ = *3.44, p*=*0.133, η*^*2*^=*0.46;* Fig. 8A), however, inactivation and overactivation of scACC-25 had opposing effects on sucrose consumption (*F*_*(2,8)*_=*21.72, p*<*0.001, η*^*2*^=*0.84;* Fig. 8B). Inactivation increased (*F*_*(1,4)*_=*13.90, p*=*0.20, η*^*2*^=*0.78 – Simple Contrast*), and overactivation decreased (*F*_*(1,4)*_=*10.79, p*=*0.030, η*^*2*^ = *0.73*) sucrose consumption, whilst neither manipulation impacted water intake (*F*<*1*; Fig. 8C).

### 3.5 Summary of results

Table 3 summarises the results from this study alongside additional results from previous studies using the same behavioural tests to provide a comprehensive overview of the effects of manipulations of marmoset scACC-25 and to place the current results into context. All results were based on groups of mixed male and female marmosets (detailed in Table 1) and there were no obvious differences in performance between the sexes at baseline (saline conditions) or following interventions (MB and DHK). There were also no obvious performance differences between marmosets at baseline or following interventions based on their precise cannulae locations along the anterior-posterior axis or dorsal-ventral axis (compare individual subject points).

**Table 3:**
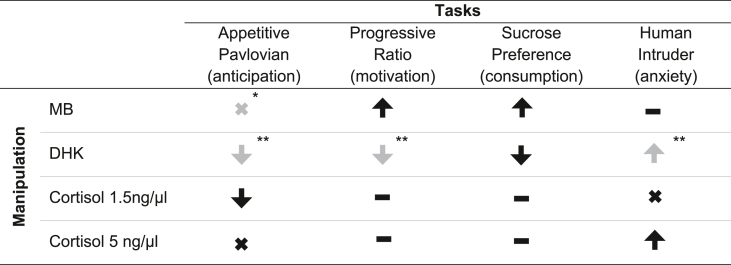
Summary of manipulations in scACC-25 in tasks used in this study.

Upward pointing arrows indicate increases, and downward pointing arrows, decreases in performance. A ‘cross’ indicates no change in behaviour and a dashed line indicates the experiment has not been carried out. Symbols in grey indicate that the results were from a previously published study. *Wallis et al. (2017), **Alexander et al. (2018).

## 4 Discussion

The present study addressed two questions. The first was whether at least some of the actions of the stress hormone cortisol were mediated via the activation of scACC-25. Results of experiment 1 showed that direct elevation of the stress hormone, cortisol, within scACC-25, resulted in a pattern of behavioural changes that mirror those seen following scACC-25 overactivation (Alexander et al., 2018). There was both a dampening of behavioural anticipatory appetitive arousal, as well as an increase in responsivity to uncertain threat. A comparable dampening of appetitive behavioural arousal was also seen following systemic administration of cortisol. The second question was regarding the extent to which scACC-25 regulated appetitive behaviours. Unlike the previously reported lack of effect of inactivation of scACC-25 on Pavlovian anticipatory arousal (Alexander et al., 2018), here inactivation did impact motivated responding (experiment 2). Specifically, inactivation increased the number of responses they made for reward and increased their consummatory behaviour. The opposing effects seen following scACC-25 overactivation, namely decreased responding for reward and decreased sucrose consumption highlights the bidirectional effects of these manipulations.

### 4.1 Cortisol infused locally into scACC-25 mirrors the behavioural effects of scACC-25 overactivation

Since heightened reactivity to uncertain threat and blunted Pavlovian anticipatory appetitive arousal is seen following infusions of either the glutamate transporter blocker DHK or cortisol into scACC-25, it supports the hypothesis that some of the effects of stress-induced cortisol release are dependent on scACC-25 activation. There appeared to be differential sensitivity of scACC-25 to the two doses of cortisol whereby blunting of anticipatory appetitive behavioural responses was induced by the low dose, 1.5 ng/μl, whilst only the higher dose of 5 ng/μl heightened responsivity to uncertain threat. When compared to systemic administration of 20 mg/kg cortisol comparable effects on reward responsivity were seen, although in both cases, peripheral or central cortisol failed to significantly reduce cardiovascular arousal during this period. Whilst overall similarity in the pattern of effects is consistent with the hypothesis that cortisol acts to heighten scACC-25 activity it is unclear why behavioural anticipatory arousal is more sensitive to cortisol compared to cardiovascular anticipatory responses. This may relate to pharmacological differences between the studies, with DHK heightening activity broadly across scACC-25, whilst cortisol may impact mineralocorticoid or glucocorticoid signalling on specific scACC-25 neurons that mediate behaviour related processes rather than those involved in the central regulation of cardiovascular activity. It should be noted though that cardiovascular changes were still in the same direction as the behavioural changes, despite not being significant.

The timing of these effects were relatively rapid, being observed within an 8-min window likely reflecting the fast acting, nongenomic effects of cortisol. The literature, overall, suggests that nongenomic actions of cortisol emerge within 5 min in brain regions such as the hippocampus (Karst et al., 2005). Rapid nongenomic effects of cortisol were also likely to be responsible for the systemic cortisol effects in which the pre-treatment time was 15 min. Certainly, intramuscular injections of cortisol in humans have been shown to alter fMRI activity as early as 7 min, disappearing by 20–27 min (Strelzyk et al., 2012). In contrast, genomic actions typically start to emerge after 1 h when the nongenomic effects have disappeared (Henckens et al., 2012). Recent evidence suggests that these rapid non-genomic effects of glucocorticoids, such as cortisol, may be through membrane-bound mineralocorticoid receptors inducing shifts in pyramidal neuron excitability (Karst and Joëls, 2023). Therefore, with the high concentration of mineralocorticoid and glucocorticoid receptors located in scACC-25 of non-human primates (Patel et al., 2000), increases in circulating cortisol, such as during periods of acute stress, may rapidly modulate behaviour via this region.

That the functional effect of cortisol infusions into scACC-25 mimic that of scACC-25 overactivation contrasts with the effects of cortisol in other frontal lobe regions, which often mimic the effects of inactivation, by disrupting higher-order mechanisms such as working memory; although the latter appear more dependent on the genomic actions of glucocorticoids (Barsegyan et al., 2010; Roozendaal et al., 2004). These contrasting findings are consistent with the anti-correlation of the dysregulated activity in these regions in depression (Fox et al., 2012).

### 4.2 Bidirectional effects of scACC-25 interventions on response breakpoint

The bidirectional effects of scACC-25 manipulation on motivation appear distinct from previously reported unidirectional effects of overactivation on Pavlovian appetitive arousal. Ceiling effects are unlikely to explain the lack of effect of inactivation on the latter since inactivation of neighbouring area 14 enhances behavioural and cardiovascular appetitive arousal in the same test (Stawicka et al., 2020). It is worth noting that the bidirectional effects seen on breaking point of the PR test were not seen on other measures of performance. Overactivation reduced overall response rate, not only increasing the latency between individual responses leading up to a reward, but also increasing the PRP. However, inactivation had no such effects on latencies but did instead increase non-rewarded licking behaviour, suggestive of an increase in reward directed Pavlovian responses. Whether these contrasting effects reflect distinct underlying mechanisms is unclear. Due to very low levels of licking outside of reward at baseline, this measure could not have been reduced following overactivation and so bi-directional effects could not have been seen. In contrast, instrumental responses were not so high as to prevent bi-directional effects from being seen.

A likely mediator of these instrumental and Pavlovian effects is the nucleus accumbens (NAc) which receives a major input from scACC-25 (Freedman et al., 2000). The NAc has been implicated in Pavlovian approach responses (Day and Carelli, 2007) and previous findings from our laboratory have identified the scACC-25 to NAc core pathway as a mediator of the blunting of Pavlovian appetitive behavioural and cardiovascular arousal induced by scACC-25 overactivation (Wood et al., 2023). The NAc has also been implicated in motivated instrumental responding, with response rate, in particular, associated with perceived effort values (Chung, 1965) and may relate to dopamine levels (Salamone et al., 2009; Sokolowski et al., 1998). Of particular relevance to scACC-25 is the finding in rodents that infralimbic cortex (ILc) modulates NAc dopamine firing activity, with inactivation heightening and overactivation reducing VTA activity (Patton et al., 2013). Whilst the functional homology of scACC-25 and ILc is far from clear, with opposing effects having been reported for the regulation of threat (Wallis et al., 2017), there is greater correspondence between scACC-25 and ILc with respect to the regulation of rewarded behaviour (Alexander et al., 2022; Patton et al., 2013). Thus, the reductions in response rate observed following scACC-25 overactivation in the current study may be the result of increased effort mediated through the accumbens dopamine pathway.

Consumption on the other hand is related to opioid signalling within the ventral striatum (Craske et al., 2016). Specifically, μ-opioid receptors in the NAc shell are associated with licking and feeding behaviour (Castro and Berridge, 2014; Cleary et al., 1996; Henderson-Redmond and Czachowski, 2014; Richard and Fields, 2016) and microinjection of a μ-opioid agonist into the NAc shell increases “liking” of sucrose solution in rodents i.e. enhancing hedonic capacity (Mitchell et al., 2018; Peciña and Berridge, 2005). In the present study scACC-25 inactivation increased food-oriented responses in the progressive ratio test but also increased sucrose consumption in the sucrose preference test. Since scACC-25 projects to both NAc subregions (Kunishio and Haber, 1994) then the effects of inactivation on consummatory responses may be mediated through alternative pathways to the NAc that mediate opioid signalling. Whilst scACC-25 overactivation did not impact licking behaviour on progressive ratio it did reduce sucrose consumption. This contrasts with our previous results where overactivation had no such effect, albeit with a higher concentration of sucrose (10%) than used here (6%) (Alexander et al., 2018). The sucrose concentration markedly alters the threshold for animals to engage with the reward (Cleary et al., 1996) therefore the most likely explanation for this discrepancy is the higher concentration produced a ceiling effect, with the lower sucrose concentration here unmasking the consummatory effects of scACC-25 overactivation. Thus, an increase in the overall numbers of responses following scACC-25 inactivation on the PR test may be the result of increased reward value as distinct from an increase in response effort that likely mediates scACC-25 overactivation effects on response rate.

### 4.3 Limitations of the study

First, the lack of direct evidence for MR- or GR-mediated actions of cortisol in scACC-25 is a limitation of experiment 1. Although, the time window suggests that the actions are likely to be MR-mediated, the higher concentration of cortisol can engage both mineralocorticoid and glucocorticoid receptors (Joëls, 2018). Second, although the role of dopaminergic system in effort and response rate, and role of opioid system in consummatory behaviour has been shown previously, the present study does not provide direct evidence for scACC's contribution to distinct mechanisms of action on motivation.

## 5 Conclusion

These findings indicate scACC-25 is an important regulator of responsivity to threat and reward. They provide important insight into the functions of this region and its relevance to our understanding of the stress-related disorders of depression and anxiety. Increased activation of scACC-25 likely accompanies the stress induced by the presence of threat and, through actions of cortisol on glucocorticoid and mineralocorticoid receptors in scACC-25, enhances behaviours that increase avoidance of the threat. Since, scACC-25 overactivation also blunts consummatory, anticipatory and motivated behaviours for reward, as does scACC-25 infusions of cortisol, it is likely that these act as adaptive responses reducing the competition between approach and avoidance responses in times of threat. However, scACC-25 appears also to be important for providing tonic inhibitory control of motivated appetitive behaviours even in the absence of explicit threat, as shown by the marked increase in responding for reward, both consummatory and instrumental, when scACC-25 was inactivated. Some of these effects may be mediated through scACC-25 projections onto distinct neurochemical circuits within the NAc, which may be revealed in future studies. What is clear is that the balance of motivated behaviours to avoid threat and approach reward is in part dependent upon activity in scACC-25 and that alterations in tonic levels of activity within scACC-25, as may occur during stress, can alter that balance.

## Funding

This research was funded by the Wellcome Trust (grant numbers 108089/Z/15/Z and 224432/Z/21/Z to ACR); and Medical Research Council (grant numbers, MR/M023990/1 and MR/V033492/1 to ACR)

## CRediT authorship contribution statement

**Rana Banai Tizkar:** Writing – review & editing, Writing – original draft, Visualization, Methodology, Formal analysis, Data curation, Conceptualization. **Lauren McIver:** Methodology. **Christian Michael Wood:** Writing – review & editing, Writing – original draft, Visualization, Methodology, Formal analysis, Data curation, Conceptualization. **Angela Charlotte Roberts:** Writing – review & editing, Writing – original draft, Visualization, Resources, Methodology, Funding acquisition, Formal analysis, Data curation, Conceptualization.

## Declaration of competing interest

The authors declare that they have no known competing financial interests or personal relationships that could have appeared to influence the work reported in this paper.

## Data Availability

I have shared the link to my data at the Attach Step Subcallosal area 25-its responsivity to the stress and its bidirectional effects on motivation (Original data) (Mendeley Data)
